# Role of helicity of α-helical antimicrobial peptides to improve specificity

**DOI:** 10.1007/s13238-014-0061-0

**Published:** 2014-05-09

**Authors:** Yibing Huang, Liyan He, Guirong Li, Naicui Zhai, Hongyu Jiang, Yuxin Chen

**Affiliations:** 1 Key Laboratory for Molecular Enzymology and Engineering of the Ministry of Education, Jilin University, Changchun, 130012 China; 2 National Engineering Laboratory for AIDS Vaccine, Jilin University, Changchun, 130012 China; 3 School of Life Sciences, Jilin University, Changchun, 130012 China; 4 The First Hospital, Jilin University, Changchun, 130021 China

**Keywords:** antimicrobial peptides (AMPs), peptide antibiotics, helicity, secondary structure, diastereomeric peptides, specificity, therapeutic index

## Abstract

A major barrier to the use of antimicrobial peptides as antibiotics is the toxicity or ability to lyse eukaryotic cells. In this study, a 26-residue amphipathic α-helical antimicrobial peptide A12L/A20L (Ac-KWKSFLKTFKSLKKTVLHTLLKAISS-amide) was used as the framework to design a series of D- and L-diastereomeric peptides and study the relationships of helicity and biological activities of α-helical antimicrobial peptides. Peptide helicity was measured by circular dichroism spectroscopy and demonstrated to correlate with the hydrophobicity of peptides and the numbers of D-amino acid substitutions. Therapeutic index was used to evaluate the selectivity of peptides against prokaryotic cells. By introducing D-amino acids to replace the original L-amino acids on the non-polar face or the polar face of the helix, the hemolytic activity of peptide analogs have been significantly reduced. Compared to the parent peptide, the therapeutic indices were improved of 44-fold and 22-fold against Gram-negative and Gram-positive bacteria, respectively. In addition, D- and L-diastereomeric peptides exhibited lower interaction with zwitterionic eukaryotic membrane and showed the significant membrane damaging effect to bacterial cells. Helicity was proved to play a crucial role on peptide specificity and biological activities. By simply replacing the hydrophobic or the hydrophilic amino acid residues on the non-polar or the polar face of these amphipathic derivatives of the parent peptide with D-amino acids, we demonstrated that this method could have excellent potential for the rational design of antimicrobial peptides with enhanced specificity.

## Introduction

In recent years, resistant superbugs have become a great concern in public health due to the extensive clinical use of classical antibiotics and prompting an urgent need for a new class of antibiotics (Oyston et al., 2009). Compared to the traditional antibiotics, cationic antimicrobial peptides (AMPs) exhibit several unique characteristics, including the ability to rapidly kill target cells, broad spectrum activity against serious antibiotic-resistant pathogens in the clinic, and the relative difficulty in selecting resistant mutants *in vitro* (Jenssen et al., 2006; Huang et al., 2010b). Therefore, AMPs have been proposed as potent candidates of a new class of antibiotics.

Nowadays, many natural and synthesized AMPs have been identified with antimicrobial activity (Shai, 1999; Shai and Oren, 2001; Wang et al., 2009a) and several action models that attempt to explain the mechanism of action, such as the “carpet” model (Shai, 1999), the “barrel-stave” model (Ehrenstein and Lecar, 1977) and the “toroidal-pore” model (Matsuzaki et al., 1995). Recently, based on the “barrel-stave” model and the “carpet” model, Chen et al. proposed a “membrane discrimination” model for AMPs whose sole target is the biomembrane, and the peptide specificity to eukaryotic or prokaryotic cells depends upon the compositional difference in the lipids of membranes (Chen et al., 2005; Chen et al., 2007). Although the precise mechanism of action of AMPs has not been fully deciphered, it is believed that the cytoplasmic membrane is the main target and the interaction with the cell membrane is the key step for all AMPs (Hancock and Rozek, 2002).

The toxicity of AMPs against eukaryotic cells is the key obstacle for their clinical application. Numerous studies have been performed to optimize their potentials for clinical applications, i.e., to improve the antimicrobial activity and to reduce the toxicity against human normal cells (Pag et al., 2004; Wang et al., 2009b; Huang et al., 2010a). In the previous study, the hydrophobicity and net charge were studied for the relationships of structure and mechanism of action of AMPs (Chen et al., 2005; Chen et al., 2007; Jiang et al., 2008). In this study, the helicity was selected to study the relationships of secondary structure and the selectivity against microbial cells of α-helical antimicrobial peptides. Two groups of D- and L-diastereomeric peptides were designed and the helicity was systematically modulated by introducing D-amino acids to replace the original L-amino acids on the non-polar face or the polar face of the α-helical antimicrobial peptides. We believe that peptide helicity plays an important role on the specificity and the toxicity of antimicrobial peptides.

## Results

### Peptide design

**Figure 1:**
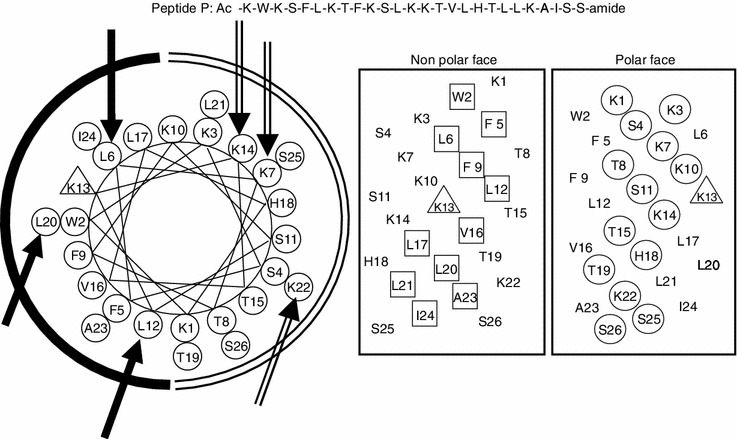
Representation of the parent peptide A12L/A20L as helical nets showing the polar/hydrophilic face (*circled residues*) and non-polar/ hydrophobic face (*boxed residues*) and helical wheel, the lysine residue at position 13 of the sequence is denoted by a *triangle*. In the helical nets, the D-amino acid substitution sites are shown in *bold* and *italic*, while in the helical wheel, three single substitution sites are shown with *solidarrows* on the non-polar face as a *solidarc* and *hollowarrows* on the polar face as an *openarc*, respectively, Ac denotes N^α^-acetyl, and amide denotes C^α^-amide. *One*-*lettercodes* are used for the amino acid residues

In this study, a 26-residue amphipathic α-helical antimicrobial peptide of A12L/A20L from the previous studies (Ac-KWKSFLKTFKSLKKTVLHTLLKAISS-amide, named as peptide P herein) with a strong α-helical structure (Chen et al., 2007) was used as a framework to design a series of D- and L-diastereomeric peptides and study the relationships of helicity and biological activities of α-helical antimicrobial peptides. The helicity was systematically reduced in various degrees by replacing L-lysine residues with D-lysine residues on the polar face as well as L-leucine residues with D-leucine residues on the non-polar face, respectively. The sequences of peptide analogs are shown in Table 1 and the helical nets and the helical wheel of peptide P are shown in Fig. 1. In order to reduce peptide helicity of peptide P to different degrees, on the polar face, positions of 7, 14 and 22 were selected to make three single D-lysine substituted peptides (K7_D_, K14_D_, K22_D_), two double D-lysine substituted peptides (K7_D_/K14_D_ and K14_D_/K22_D_), and one triple D-lysine substituted analog (K7_D_/K14_D_/K22_D_), respectively. In order to study the effect of helicity change on peptide biological activities, three peptides with the corresponding 4, 5 and 6 D-lysine substitutions (K7_D_/K10_D_/K14_D_/K22_D_, K3_D_/K7_D_/K10_D_/K14_D_/K22_D_, and K1_D_/K3_D_/K7_D_/K10_D_/K14_D_/K22_D_, respectively) were designed to further reduce the helicity of peptide P. In contrast, on the non-polar face, positions of 6, 12 and 20 were selected according to the similar design with those on the polar face to make single and multiple D-leucine substituted peptides (from L6_D_ to L6_D_/L12_D_/L17_D_/L20_D_/L21_D_).

**Table 1:** Design and sequence of α-helical antimicrobial peptides

Group	Peptide	Amino acid sequence*
Parent	P	Ac-K-W-K-S-F-L-K-T-F-K-S-L-K-K-T-V-L-H-T-L-L-K-A-I-S-S-amide
Polar face group	K7_D_	Ac-K-W-K-S-F-L-***K***-T-F-K-S-L-K-K-T-V-L-H-T-L-L-K-A-I-S-S-amide
	K14_D_	Ac-K-W-K-S-F-L-K-T-F-K-S-L-K-***K***-T-V-L-H-T-L-L-K-A-I-S-S-amide
	K22_D_	Ac-K-W-K-S-F-L-K-T-F-K-S-L-K-K-T-V-L-H-T-L-L-***K***-A-I-S-S-amide
	K7_D_/K14_D_	Ac-K-W-K-S-F-L-***K***-T-F-K-S-L-K-***K***-T-V-L-H-T-L-L-K-A-I-S-S-amide
	K14_D_/K22_D_	Ac-K-W-K-S-F-L-K-T-F-K-S-L-K-***K***-T-V-L-H-T-L-L-***K***-A-I-S-S-amide
	K7_D_/K14_D_/K22_D_	Ac-K-W-K-S-F-L-***K***-T-F-K-S-L-K-***K***-T-V-L-H-T-L-L-***K***-A-I-S-S-amide
	K7_D_/K10_D_/K14_D_/K22_D_	Ac-K-W-K-S-F-L-***K***-T-F-***K***-S-L-K-***K***-T-V-L-H-T-L-L-***K***-A-I-S-S-amide
	K3_D_/K7_D_/K10_D_/K14_D_/K22_D_	Ac-K-W-***K***-S-F-L-***K***-T-F-***K***-S-L-K-***K***-T-V-L-H-T-L-L-***K***-A-I-S-S-amide
	K1_D_/K3_D_/K7_D_/K10_D_/K14_D_/K22_D_	Ac-***K***-W-***K***-S-F-L-***K***-T-F-***K***-S-L-K-***K***-T-V-L-H-T-L-L-***K***-A-I-S-S-amide
Non-polar face group	L6_D_	Ac-K-W-K-S-F-***L***-K-T-F-K-S-L-K-K-T-V-L-H-T-L-L-K-A-I-S-S-amide
	L12_D_	Ac-K-W-K-S-F-L-K-T-F-K-S-***L***-K-K-T-V-L-H-T-L-L-K-A-I-S-S-amide
	L20_D_	Ac-K-W-K-S-F-L-K-T-F-K-S-L-K-K-T-V-L-H-T-***L***-L-K-A-I-S-S-amide
	L6_D_/L12_D_	Ac-K-W-K-S-F-***L***-K-T-F-K-S-***L***-K-K-T-V-L-H-T-L-L-K-A-I-S-S-amide
	L12_D_/L20_D_	Ac-K-W-K-S-F-L-K-T-F-K-S-***L***-K-K-T-V-L-H-T-***L***-L-K-A-I-S-S-amide
	L6_D_/L12_D_/L20_D_	Ac-K-W-K-S-F-***L***-K-T-F-K-S-***L***-K-K-T-V-L-H-T-***L***-L-K-A-I-S-S-amide
	L6_D_/L12_D_ /L17_D_/L20_D_	Ac-K-W-K-S-F-***L***-K-T-F-K-S-***L***-K-K-T-V-***L***-H-T-***L***-L-K-A-I-S-S-amide
	L6_D_/L12_D_ /L17_D_/L20 _D_ /L21_D_	Ac-K-W-K-S-F-***L***-K-T-F-K-S-***L***-K-K-T-V-***L***-H-T-***L***-***L***-K-A-I-S-S-amide

* One-letter codes are used for the amino acid residues; the bold italic letters denote the substituting D-amino acids of the peptide P, all other amino acids are L-amino acids

### Peptide secondary structure

Table 2 shows the molar ellipticity values at different environments and the relative helicity of peptide analogs. It is well studied that D-amino acids show disruptive ability on α-helical peptide composed of all L-amino acids (Shai and Oren, 1996; Chen et al., 2005; Prenner et al., 2005). In this study, all peptides showed the typical α-helical structure with double minima at 208 nm and 222 nm in a hydrophobic environment of 50% TFE to mimic the cell membrane, while all the D-amino acid substituted peptides exhibited reduced helical structures in a hydrophilic environment of KP buffer. The helicity of peptide was strongly influenced by the number of the substituted D-amino acids and gradually decreased with the increasing numbers of D-amino acid substitutions both on the polar face and the non-polar face. The relative helicity of peptide analogs ranged from 100% (Peptide P) to 48.6% (K1_D_/K3_D_/K7_D_/K10_D_/K14_D_/K22_D_) with the D-lysine substitutions on the polar face and to 33.2% (L6_D_/L12_D_/L17_D_/L20_D_/L21_D_) with the D-leucine substitutions on the non-polar face.

**Table 2:** Biophysical data of the peptide analogs

Peptides^a^	t_R_ (min)^b^	Benign^c^		50% TFE^d^	
	25°C	[θ]_222_	% helix^e^	[θ]_222_	% helix^e^
P	46.9	−14550	36.66	−39700	100.00
K7_D_	44.1	−6050	15.22	−30900	77.77
K14_D_	43.5	−15550	39.16	−35250	88.72
K22_D_	43.3	−8400	21.14	−33950	85.48
K7_D_/K14_D_	40.9	−8750	22.02	−28450	71.65
K14_D_/K22_D_	40.1	−6350	15.95	−32150	81.00
K7_D_/K14_D_/K22_D_	37.9	−5000	12.59	−26350	66.33
K7_D_/K10_D_/K14_D_/K22_D_	35.5	−7350	22.48	−19400	48.84
K3_D_/K7_D_/K10_D_/K14_D_/K22_D_	34.6	−4750	14.59	−21400	53.99
K1_D_/K3_D_/K7_D_/K10_D_/K14_D_/K22_D_	34.6	−5800	17.74	−19300	48.61
L6_D_	44.6	−8500	21.45	−39550	99.61
L12_D_	42.9	−8350	21.01	−37750	95.08
L20_D_	42.5	−6900	17.42	−36900	92.90
L6_D_/L12_D_	40.8	−6800	17.14	−29050	73.13
L12_D_/L20_D_	37.8	−5550	13.93	−30050	75.69
L6_D_/L12_D_/L20_D_	37.4	−4950	12.45	−28450	71.66
L6_D_/L12_D_ /L17_D_/L20_D_	36.3	−4050	12.46	−15050	37.88
L6_D_/L12_D_ /L17_D_/L20_D_ /L21_D_	34.8	−3600	10.97	−13150	33.20

^a^Peptides are ordered by relative hydrophobicity

^b^*t*_R_ (min) denotes the retention time at 25°C by RP-HPLC

^c^The mean residue molar ellipticities, [θ]_222_ (degree cm^2^ dmol^−1^) at wavelength 222 nm were measured at 25°C in KP buffer (100 mmol/L KCl, 50 mmol/L PO_4_, pH 7.0)

^d^The mean residue molar ellipticities, [θ]_222_ (degree cm^2^ dmol^−1^) at wavelength 222 nm were measured at 25°C in KP buffer with 50% TFE

^e^The helical content (in percentage) of a peptide relative to the molar ellipticity value of peptide P in 50% TFE

Furthermore, the position of the substituted D-amino acids may influence the helicity of peptide. From Table 2, it is clear to see that N-terminal amino acids in the peptide P sequence may be more important to stabilize the helical structure since the relative helicity values of peptide K7_D_/K14_D_ (71.7%) and L6_D_/L12_D_ (73.1%) were lower than those of the peptide K14_D_/K22_D_ (81%) and L12_D_/L20_D_ (75.7%) respectively. It is interesting to see that, single L-lysine on the polar face had more important role to sustain the helical structure than L-leucine on the non-polar face due to the relative helicity values of single D-amino acid substituted peptides K7_D_ (77.8%), K14_D_ (88.7%) and K22_D_ (85.5%) were less than those of L6_D_ (99.6%), L12_D_ (95.1%) and L20_D_ (92.9%), respectively. However, when comes to multiple substitutions, 4 D-leucine substitutions on the peptide showed a stronger disruptive effect on α-helical structure than 6 D-lysine substitutions (48.61% of helicity for peptide K1_D_/K3_D_/K7_D_/K10_D_/K14_D_/K22_D_ and 37.88% for peptide L6_D_/L12_D_/L17_D_/L20_D_, respectively), showing the important effect of hydrophobicity on sustaining peptide secondary structure.

### Peptide hydrophobicity

RP-HPLC retention behaviors of peptides are highly sensitive to the conformational status upon interacting with the hydrophobic environments of the column matrix and are widely utilized to represent the relative hydrophobicity of peptides (Zhou et al., 1990; Chen et al., 2002). In this study, the hydrophobicity of peptides was determined by RP-HPLC. The hydrophobicity difference of peptides caused by D-amino acid substitutions was mainly due to the change on the continuity of the hydrophobic/hydrophilic face of the helical structure, since the side chain hydrophobicity of D- and L-amino acid enantiomers is exactly the same. From Table 2, the hydrophobicity of peptides (as expressed by RP-HPLC retention time *t*_R_) decreased gradually with the increasing numbers of D-amino acid substitutions on both the polar face and the non-polar face of peptide analogs while *t*_R_ ranging from 44.1 min (K7_D_) to 34.6 min (K1_D_/K3_D_/K7_D_/K10_D_/K14_D_/K22_D_) on the polar face and from 44.6 min (L6_D_) to 34.8 min (L6_D_/L12_D_/L17_D_/L20_D_/L21_D_) on the non-polar face, respectively. This order was the same as the aforementioned order of helicity and confirmed to the previous results that peptide helicity was correlated with peptide hydrophobicity (Huang et al., 2010a; Huang et al., 2011). Thus, D-amino acid substitutions not only affect the helicity of peptides,but also affect the hydrophobicity of peptides by changing the continuity of the hydrophobic and hydrophilic faces of α-helical structure.

### Hemolytic activity

The minimal hemolytic concentration (MHC) of the peptide analogs against human erythrocytes was determined as a major measurement of peptide toxicity toward normal cells (Table 3). Compared to the peptide P (MHC 5.2 μmol/L), the peptide hemolytic activity significantly decreased to no detectable hemolysis at the concentration of 325.2 μmol/L by substituting D-amino acid both on the non-polar face and the polar face of the parent peptide. Compared to the single substituted peptides and the double substituted peptides, the peptides with multiple substitutions both on the polar face and on the non-polar face showed lower toxicity toward normal red blood cells. This phenomenon is similar to that of helicity and hydrophobicity. That is, due to the substitutions of D-amino acids, the helicity of peptides was disrupted and results in the decrease of peptide hydrophobicity and the hemolytic activity.

**Table 3:** Antimicrobial (MIC) and hemolytic (MHC) activities of peptide analogs against Gram-negative bacteria and human red blood cells

Peptides^a^	MHC^b^ (µmol/L)	MIC^c^ (µmol/L)		GM^d^	Therapeutic index^e^	Fold^f^
		*E. coli* ATCC25922	*P. aeruginosa* ATCC27853			
P	5.2	2	8	4.0	1.3	1.0
K7_D_	10.41	1	2	1.4	7.4	5.7
K14_D_	5.2	1	2	1.4	3.7	2.8
**K22**_**D**_^g^	**20.81**	**1**	**1**	**1.0**	**20.8**	**16.0**
K7_D_/K14_D_	20.81	2	2	2.0	10.4	8.0
**K14**_**D**_/**K22**_**D**_	**20.81**	**1**	**1**	**1.0**	**20.8**	**16.0**
K7_D_K14_D_K22_D_	81.31	8	2	4.0	20.3	15.6
K7_D_/K10_D_/K14_D_/K22_D_	325.2	64	8	22.6	14.4	11.1
K3_D_/K7_D_/K10_D_/K14_D_/K22_D_	>325.2	8	32	16.0	40.7	31.3
K1_D_/K3_D_/K7_D_/K10_D_/K14_D_/K22_D_	>325.2	125	32	63.3	10.3	7.9
L6_D_	10.41	2	1	1.4	7.4	5.7
L12_D_	20.81	1	1	1.0	20.8	16.0
L20_D_	20.81	1	1	1.0	20.8	16.0
L6_D_/L12_D_	20.81	4	2	2.8	7.4	5.7
**L12**_**D**_/**L20**_**D**_	**81.31**	**2**	**2**	**2.0**	**40.7**	**31.3**
**L6**_**D**_/**L12**_**D**_/**L20**_**D**_	**162.61**	**4**	**2**	**2.8**	**57.5**	**44.2**
L6_D_/L12_D_/L17_D_/L20_D_	81.3	32	8	16.0	5.1	3.9
L6_D_/L12_D_/L17_D_/L20_D_/L21_D_	>325.2	16	4	8.0	81.3	62.5

^a^Peptides are ordered by relative hydrophobicity

^b^Hemolytic activity (minimal hemolytic concentration) was determined on human red blood cells after incubating with peptides for 1 h (hRBC). When no hemolytic activity was observed at 325.2 μmol/L, a value of 650.4 μmol/L was used for the calculation of the therapeutic index

^c^Antimicrobial activity (minimal inhibitory concentration) was determined as the minimal concentration of peptide to inhibit microbial growth

^d^*GM* denotes the geometric mean of MIC values from two microbial strains in this table

^e^Therapeutic index = MHC (μmol/L)/geometric mean of MIC (μmol/L), larger values indicate greater antibacterial specificity

^f^The fold improvement in the therapeutic index was determined as relative to that of parent peptide P

^g^The bold data represent the leading peptide analogs with great specificity improvement

### Antimicrobial activity

The antimicrobial activities of peptide analogs were determined against both Gram-negative and Gram-positive bacterial strains. The results are showed in Table 3 and Table 4. The geometric mean MIC (minimal inhibitory concentration) values of microbial strains were calculated to provide an overall evaluation of antimicrobial activity against Gram-negative or Gram-positive bacteria, respectively. Generally, the peptides with more D-amino acid substitutions exhibited lower antimicrobial activity compared to the analogs with single or double D-amino acid substitutions. In this study, by substituting D-amino acids on the polar or the non-polar face of the parent peptide, we have dramatically improved peptide antimicrobial activities, especially against Gram-negative bacteria (Tables 3 and 4). However, when introducing relatively more D-amino acids (4 and more D-amino acids), the antimicrobial activities decreased significantly against both Gram-negative and Gram-positive bacteria. Compared to peptide P, the single and double amino acid substituted peptides (on both the polar and the non-polar faces) exhibited close antimicrobial activities with MIC values from 1–2 μmol/L against Gram-negative and Gram-positive bacteria. However, along with the further increasing of D-amino acid substitutions, peptide analogs exhibited significant lower antimicrobial activity with MIC values ranging from 4–125 μmol/L. It is clear to see that peptides with higher helicity generally exhibited stronger antimicrobial activities, which indicates the importance of helicity of peptides during the mechanism of action against microbial strains.

**Table 4:** Antimicrobial (MIC) and hemolytic (MHC) activities of peptide analogs against Gram-positive bacteria and human red blood cells

Peptides^a^	MHC^b^ (µmol/L)	MIC^c^ (µmol/L)			Therapeutic index^e^	Fold^f^
		*S. aureus* ATCC25923	*B. subtilis* ATCC49619	GM^d^		
P	5.2	4	1.0	2.0	2.6	1.0
K7_D_	10.41	4	0.25	1.0	10.4	4.0
K14_D_	5.2	2	0.25	0.7	7.3	2.8
**K22**_**D**_^g^	**20.81**	**4**	**0.5**	**1.4**	**14.8**	**5.7**
K7_D_/K14_D_	20.81	8	0.5	2.0	10.4	4.0
K14_D_/K22_D_	20.81	8	0.5	2.0	10.4	4.0
K7_D_K14_D_K22_D_	81.31	32	0.5	4.0	20.3	7.8
K7_D_/K10_D_/K14_D_/K22_D_	325.2	>125	0.5	11.2	29.1	11.2
K3_D_/K7_D_/K10_D_/K14_D_/K22_D_	>325.2	>125	2.0	22.4	29.1	11.2
K1_D_/K3_D_/K7_D_/K10_D_/K14_D_/K22_D_	>325.2	>125	0.5	11.2	58.2	22.4
L6_D_	10.41	4	1.0	2.0	5.2	2.0
L12_D_	20.81	4	1.0	2.0	10.4	4.0
L20_D_	20.81	4	1.0	2.0	10.4	4.0
L6_D_/L12_D_	20.81	8	1.0	2.8	7.4	2.8
**L12**_**D**_/**L20**_**D**_	**81.31**	**8**	**0.25**	**1.4**	**57.7**	**22.2**
**L6**_**D**_ **/L12**_**D**_ **/L20**_**D**_	**162.61**	**32**	**0.25**	**2.8**	**57.5**	**22.1**
L6_D_/L12_D_/L17_D_/L20_D_	81.3	>125	0.5	11.2	7.3	2.8
L6_D_/L12_D_/L17_D_/L20_D_/L21_D_	>325.2	125	0.5	7.9	82.2	31.6

^a^Peptides are ordered by relative hydrophobicity

^b^Hemolytic activity (minimal hemolytic concentration) was determined on human red blood cells after incubating with peptides for 1 h (hRBC). When no hemolytic activity was observed at 325.2 μmol/L, a value of 650.4 μmol/L was used for the calculation of the therapeutic index

^c^Antimicrobial activity (minimal inhibitory concentration) was determined as the minimal concentration of peptide to inhibit microbial growth. When no antimicrobial activity was observed at 125 μmol/L, a value of 250 μmol/L was used for the calculation of the therapeutic index

^d^*GM* denotes the geometric mean of MIC values from two microbial strains in this table

^e^Therapeutic index = MHC (μmol/L)/geometric mean of MIC (μmol/L), larger values indicate greater antibacterial specificity

^f^The fold improvement in the therapeutic index was determined as relative to that of parent peptide P

^g^The bold data represent the leading peptide analogs with great specificity improvement

### Peptide specificity (therapeutic index)

Therapeutic index is a widely employed parameter to represent the specificity of antimicrobial reagents. It is calculated by the ratio of MHC (hemolytic activity) and MIC (antimicrobial activity) and larger values in therapeutic index indicate greater antimicrobial specificity. From Tables 3 and 4, it is clear that, with the D-amino acid substitutions on the polar or the non-polar face, the specificity of peptide P against both Gram-negative and Gram-positive bacteria has been significantly improved. For example, with D-lysine substitution on the polar face, the therapeutic index of peptide P against Gram-negative bacteria was improved to 20.8 (peptides K22_D_ and K14_D_/K22_D_), which is a 16-fold improvement; in contrast, by substituting D-leucine on the non-polar face, we improved the therapeutic index of peptide P against Gram-negative bacteria to 57.5 (peptide L6_D_/L12_D_/L20_D_), which is a 44.2-fold improvement (Table 3). In Table 4, with D-lysine substitution on the polar face, the therapeutic index of peptide P against Gram-positive bacteria was increased to 14.8 (peptide K22_D_), which is a 5.7-fold improvement compared to the parent peptide; in contrast, with D-leucine substitution on the non-polar face, the therapeutic index was improved 22.2-fold (57.7 for peptide L12_D_/L20_D_). Compared to the peptides with D-amino acid substitutions on the polar face, the peptides with D-amino acid substitutions on the non-polar face displayed higher specificity against both Gram-negative and Gram-positive bacteria due to their lower mammalian cell toxicity. Although peptide L6_D_/L12_D_/L17_D_/L20_D_/L21_D_ showed the best therapeutic index values against Gram-negative and Gram-positive bacteria, due to its poor antimicrobial activities, it was not considered as the best leading compound on specificity.

### Scanning electron microscopy

**Figure 2:**
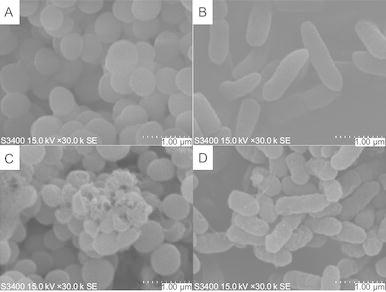
**Effect of peptide L12**_D_**/L20**_D_**on the surface of negatively-stained*S. aureus*(left) and*P. aeruginosa*(right) by scan electron microscopy**. Untreated bacterial cells were shown in panels *A* and *B*. Treated bacterial cells with peptide L12_D_/L20_D_ revealed disrupted cell membranes in *panelsC* and *D*

*Pseudomonas aeruginosa* and *Staphylococcus aureus* were used as the representatives of Gram-negative and Gram-positive bacteria, respectively, to examine the morphologic changes of cell surface before and after interacting with our leading peptide L12_D_/L20_D_ by scanning electron microscopy at 30K magnification. As showed in Fig. 2, the untreated control samples displayed smooth surface for both Gram-negative and Gram-positive bacteria (Fig. 2A and 2B); in contrast, after treated with peptide L12_D_/L20_D_ for 2 h, all bacteria showed significant surface damaging phenomena with surface wrinkling, roughening and leaking (Fig. 2C and 2D).

### Interaction of peptides with liposomes

**Figure 3:**
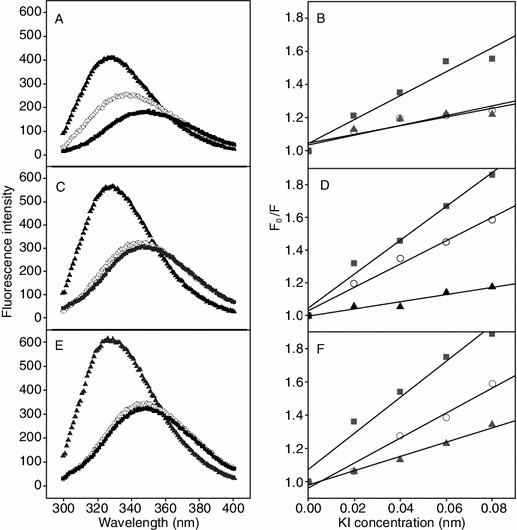
Fluorescence emission spectra (*left*) and Stern-Volmer plot (*right*) of peptides with various liposome models at 25°C. Stern-Volmer plots were obtained by the sequential addition of the fluorescence quencher KI. Results of three peptides were plotted as follows: parent peptide P in *PanelsA* and *B*, peptide K22_D_ in *PanelsC* and *D*, and peptide L12_D_/L20_D_ in *PanelsE* and *F*, respectively. HEPES buffer, PC/cholesterol lipsomes and PC/PG lipsomes were presented by *solidsquares*, *hollowcircles* and *solidtriangles*, respectively

Large unilamellar vesicles (LUV) were prepared with PC/cholesterol (8:1, *w*/*w*) and PC/PG (7:3, *w*/*w*) to mimic zwitterionic eukaryotic membrane and anionic prokaryotic membrane, respectively, and to investigate the specificity of peptides interacting with different model membranes. The fluorescence emission of the tryptophan residue was used to monitor the binding of peptides to liposomes, since fluorescence of the tryptophan residue is sensitive to different environments. The fluorescence emission maxima of peptides exhibited a blue shift and a marked increase in emission intensity when the peptides with tryptophan residue inserted into a hydrophobic environment, such as the hydrophobic core of cytoplasmic membrane (Zhang et al., 1999). Peptides P, K22_D_ and L12_D_/L20_D_ were selected to testify peptide specificity when interacting with different types of membranes. As shown in Fig. 3, it is clear that the fluorescence emission maxima of all three peptides exhibited a blue shift about 20 nm and the marked increases in emission intensity in PC/PG lipsomes compared to those in HEPES buffer (Fig. 3A, 3C and 3E), which indicates that the peptides inserted deeper into a more hydrophobic environment when interacting with prokaryotic type of membrane, thus there was a stronger interaction between peptides and prokaryotic cell membrane. In contrast, when interacting with PC/cholesterol lipsomes, only peptide P exhibited blue shift about 10 nm, while the fluorescence of peptides K22_D_ and L12_D_/L20_D_ showed the similar fluorescence emission compared to those in HEPES buffer, hence the interaction of peptides with the zwitterionic eukaryotic membrane are much weaker than with prokaryotic membrane. In addition, D-amino acid substitutions seem to have preventive effects on peptides entering into the hydrophobic core of membrane.

In order to further study the interaction of peptide with the membrane-mimicking environments, the water soluble quencher KI was used and added to peptides in different liposomes. The fluorescence intensity will be significantly reduced when the peptide containing a tryptophan residue exposed into the hydrophilic environment due to that the tryptophan residue was accessible to the aqueous quencher. As shown in Fig. 3, it is clear that KI quenched the intensity of fluorescence of tryptophan to different degrees when the peptides interacting with different types of membranes (Fig. 3B, 3D and 3F). Compared to the anionic prokaryotic membrane, the significant quenching of fluorescence intensity of the peptides in the zwitterionic eukaryotic membrane was observed, indicating that peptides did not insert into the deep hydrophobic core of eukaryotic membrane and were reachable to the water soluble quencher KI, thus resulted in the more tilted lines in Fig. 3.

## Discussion

In this study, helicity of peptide P was systematically modulated by introducing D-amino acids to replace the original L-amino acids on the non-polar face or the polar face of the helix. In order to minimize the hydrophobicity influence of amino acid substitution, D-amino acid was used to replace L-amino acid both on the non polar face and polar face and design a series of D- and L-diastereomeric peptides. In addition, acetylation of the N-terminal and amidation of the C-terminal and DL-diastereomeric structure of peptide could slow or eliminate proteolytic degradation and improve the activity or stability of peptides (Brinckerhoff et al., 1999; Papo et al., 2003; Nguyen et al., 2010).

**Figure 4:**
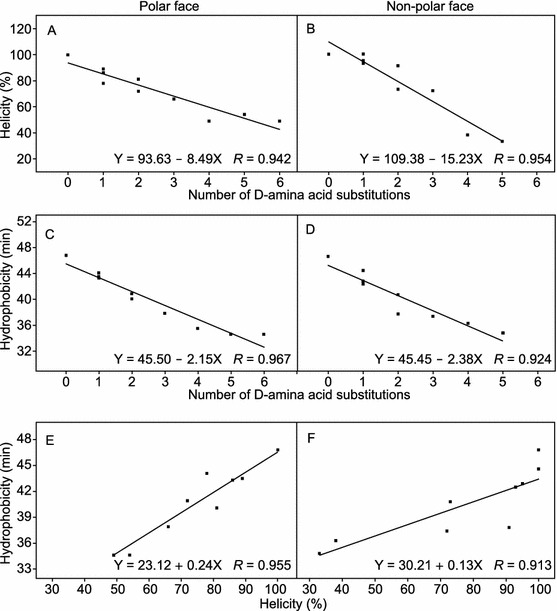
Relationships of peptide helicity, hydrophobicity with the numbers of D-amino acid substitutions. The experimental data from Table 2 and least squares fit analysis were used. The results showed correlations of helicity and the number of D-amino acid substitutions with *R* = 0.942 on the polar face (A) and *R* = 0.954 on the non-polar face (B); correlations of hydrophobicity and the number of D-amino acid substitutions with *R* = 0.967 on the polar face (C) and *R* = 0.924 on the non-polar face (D); correlations between hydrophobicity and helicity with *R* = 0.955 on the polar face (E) and *R* = 0.913 on the non-polar face (F)

As shown in Fig. 4, helicity of peptide in the hydrophobic environment and hydrophobicity of peptide exhibited excellent linear correlations with the numbers of D-amino acid substitutions both on the polar face (*R* values of 0.942 and 0.967, respectively) (Fig. 4A and 4C) and on the non-polar face (*R* values of 0.954 and 0.924, respectively) (Fig. 4B and 4D). The helicity reducing of the peptides can be attributed to that D-amino acid substitutions disrupted α-helical structure of peptides and the hydrophobicity difference of peptides caused by D-amino acid substitutions was mainly due to the change on the continuity of the hydrophobic/hydrophilic face of the helical structure, since the side chain hydrophobicity of D- and L-amino acid enantiomers are exactly the same (Chen et al., 2002; Chen et al., 2005). From Fig. 4E and 4F, it is clear that the hydrophobicity and the helicity in 50% TFE of the peptides with D-amino acid substitutions on the polar face or the non-polar face are linearly correlated with R values of 0.955 and 0.913, respectively. These results are consistent with the linear relationships of hydrophobicity and helicity of amphipathic helical anticancer peptides in the previous studies (Chen et al., 2005; Huang et al., 2011; Huang et al., 2012).

From Table 3 and Table 4, it is clear that both the hemolytic activities and the antimicrobial activities of peptides generally decreased along with the increasing of the numbers of D-amino acid substitutions on both the polar face and the non-polar face of peptides, but the degree is different. Compared to the antimicrobial activities, D-amino acid substitutions have more influence on the hemolytic activities. Thus, the therapeutic index values are not exactly linearly correlated with the increasing numbers of D-amino acid substitutions. We think this may be due to the different action mechanism of peptides against the prokaryotic cells and eukaryotic cells based on their different composition of cell membranes. In this study, several peptides with amino acid substituted peptides on the polar face or the non-polar face, particularly peptides K22_D_, K14_D_/K22_D_, L12_D_/L20_D_ and L6_D_/L12_D_/L20_D_ exhibit the strongest antimicrobial activity and the highest therapeutic index. The specificity of peptide P was improved about 44.2-fold and 22.2-fold against Gram-negative and Gram-positive bacteria, respectively. These results prove that hydrophobicity and helicity are crucial parameters for biological activities of α-helical antimicrobial peptides and are consistent with the previous studies of α-helical anticancer peptides (Huang et al., 2011; Huang et al., 2012). In addition, the approach of making D- and L-diastereomeric peptides was demonstrated as an applicable approach to improve the specificity of antimicrobial peptides which is consistent with the previous reports using model peptides (Oren and Shai, 1997; Hong et al., 1999; Papo et al., 2002).

From the results of tryptophan fluorescence and quenching experiments, the similar conclusions can be drawn that peptide specificity could be improved by the modulation helicity and hydrophobicity. Compared to the interaction with eukaryotic membrane, all peptides in this study exhibited stronger interaction with anionic prokaryotic membrane, showing blue shifts about 20 nm, marked increases in emission intensity and lower quenching effect, indicating the deeper insertion into prokaryotic membrane, and resulting in the stronger antimicrobial activity. The scanning electron microscopy results also demonstrated that all bacteria treated with peptide L12_D_/L20_D_ showed significant membrane damaging effect. In contrast, for zwitterionic eukaryotic membrane, the D- and L-diastereomeric peptides, except the parent peptide P, displayed weaker interaction with mimic zwitterionic membranes, showing lower cytotoxicity against normal cells. This is consistent well with the proposed “membrane discrimination mechanism” (Chen et al., 2005; Chen et al., 2007), Thus, based on the “membrane discrimination mechanism”, peptides use different mechanisms when interacting with prokaryotic and eukaryotic membranes, which giving us an opportunity to optimize peptide specificity and to develop peptides as promising therapeutics for clinical practices. Peptide specificity can be improved by the modulation of suitable D-amino acids on the polar face or the non-polar face of helix to form D- and L-diastereomeric peptides.

In summary, showing excellent correlation with peptide hydrophobicity, peptide helicity displays a critical role on the antimicrobial activity of α-helical antimicrobial peptides. The helicity and hydrophobicity of peptide can be modulated by D-amino acid substitution approach to form D- and L-diastereomeric peptides. In addition, D- and L-diastereomeric peptides particularly for the peptides with D-amino acid substitutions on the non-polar face displayed stronger antimicrobial activity and lower cell toxicity against normal cells. Due to the different lipid compositions between prokaryotic and eukaryotic cytoplasmic membranes, peptides exhibited high specificity against bacterial cells, which providing great opportunities to develop peptides as promising therapeutics in clinical practices.

## Materials and methods

### Peptide synthesis and purification

Peptides synthesis were carried out by solid phase peptide synthesis using Fmoc (9-fluorenyl-methoxycar-bonyl) chemistry and Rink amide 4-methylbenzhydrylamine resin (MBHA resin; 0.8 mmol/g), as described previously (Chen et al., 2005; Huang et al., 2010a). The crude peptides were purified by preparative Shimadzu LC-6A high-performance liquid chromatography (HPLC), using a Zorbax 300 SB-C_8_ column (250 × 9.4-mm ID, 6.5-mm particle size, 300-Å pore size; Agilent Technologies) with a linear AB gradient (0.1% acetonitrile/min) at a flow rate of 2 mL/min, while eluent A was 0.1% aqueous trifluoroacetic acid (TFA) in water, and eluent B was 0.1% TFA in acetonitrile. Peptide samples were analyzed on a Shimadzu LC-20A HPLC. Runs were performed on a Zorbax 300 SB-C_8_ column (150 × 4.6-mm ID, 5-mm particle size, 300-Å pore size) from Agilent Technologies, using a linear AB gradient (1% acetonitrile/min) and a flow rate of 1 mL/min, in which eluent A was 0.1% aqueous TFA and eluent B was 0.1% TFA in acetonitrile. The peptides were further characterized by mass spectrometry and amino acid analysis (Lee et al., 2003; Mant et al., 2003).

### Characterization of helical structure

The mean residue molar ellipticities of the peptides were determined by circular dichroism (CD) spectroscopy of J-810 spectropolarimeter (Jasco, JAPAN) with a 0.02-cm path length quartz cuvette at 25°C as described previously (Huang et al., 2010a). The concentration of 75 μmol/L peptides was measured in benign buffer (50 mmol/L KH_2_PO_4_/K_2_HPO_4_, 100 mmol/L KCl, pH 7) or benign buffer with 50% TFE at 25°C. The mean residue molar ellipticities were calculated by the equation [θ] = θ/10lC_M_n (Chen et al., 2005) and θ is the ellipticity in millidegrees, l is the optical path length of the cuvette in centimeters, C_M_ is the peptide concentration in mole/liter, and n is the number of residues in the peptide. The values of mean residue molar ellipticities of the peptide analogs at 222 nm were used to determine the relative helicity of the peptides.

### Measurement of antibacterial activity (MIC)

Minimal inhibitory concentrations (MIC) were determined using a broth dilution method (Stark et al., 2002) and four bacterial strains were used in this study including two Gram-negative bacterial strains of *Escherichia coli* ATCC25922, *Pseudomonas aeruginosa* ATCC27853 and two Gram-positive bacterial strains of *Staphylococcus aureus* ATCC25923, *Bacillus subtilis* ATCC6633. Briefly, bacteria were grown overnight at 37°C in Mueller-Hinton (MH) broth, diluted in the same medium and transferred into 96-well microtiter plates (90 μL/well). Peptides were serially diluted by 0.2% bovine serum albumin containing 0.01% acetic acid and added to the microtiter plates in a volume of 10 μL of each well to give a final concentration of 5 × 10^5^ CFU/mL. MICs were determined as the lowest peptide concentration that inhibited bacterial growth after incubation for 24 h at 37°C.

### Measurement of hemolytic activity (MHC)

Peptide samples were serially diluted by PBS in 96 well plates (round bottom, Corning No. 3879) to give a volume of 70 μL sample solution in each well. Human erythrocytes anticoagulated by EDTAK were collected by centrifugation (1000 ×*g*) for 5 min, and washed twice by PBS, then diluted to a concentration of 2% in PBS. 70 μL of 2% erythrocytes were added to each well to give a final concentration of 1% human erythrocytes in each well and plates were incubated at 37°C for 1 h. The plates were then centrifuged for 10 min at 3000 rpm (800 ×*g*) and supernatant (90 μL) was transferred to a 96-well plate (flat bottom, Corning No. 3599). The release of hemoglobin was determined by measuring the absorbance of the supernatant at 578 nm. The hemolytic activity was determined as the minimal peptide concentration that caused hemolysis (minimal hemolytic concentration, MHC). Erythrocytes in PBS and distilled water were used as the control of 0 and 100% hemolysis, respectively.

### Calculation of therapeutic index (MHC/MIC ratio)

Therapeutic index values were determined by the ratio of MHC/MIC, indicating the specificity of peptides against bacterial and eukaryotic cells, respectively. When there was no hemolytic activity at 325.2 µmol/L, a minimal hemolytic concentration of 650.4 µmol/L was used to calculate the therapeutic index. In contrast, for the antimicrobial activity, 650.4 µmol/L would be used if there was no activity at the upper limit value of MIC 325.2 µmol/L.

### Field emission-scanning electron microscopy (FE-SEM) analysis of bacterial cells

Bacterial cells of *Pseudomonas aeruginosa* and *Staphylococcus aureus* were cultured in Mueller-Hinton (MH) broth to its log growth-phase at 37°C under constant shaking at 180 rpm, respectively. Microorganism was harvested by centrifugation for 5 min at 4000 rpm, washed twice with 10 mmol/L PBS and re-suspended. 5 × 10^5^ cells were incubated at 37°C for up to 2 h with antimicrobial peptides L12_D_/L20_D_ at the concentration of 20 µmol/L (a concentration above the MIC of the peptide). Controls were run without peptides. Cells were fixed with 2.5% (*w*/*v*) glutaraldehyde in PBS, and then the cells were extensively washed with PBS and dehydrated with a gradation of ethanol concentrations. After critical point drying and gold coating, the samples were observed using a Hitachi S-3400N instrument (Wiradharma et al., 2011; Chen et al., 2012).

### Preparation of liposomes

Large unilamellar vesicles (LUV) were prepared with PC/cholesterol (8:1 *w*/*w*) and PC/PG (7:3 *w*/*w*) using the freeze-thaw method as described previously (Mayer et al., 1985; Zhang et al., 1999) followed by extrusion through 0.1 μm double-stacked nuclepore filters using an Mini-Extruder (Avanti Polar Lipids, Inc.).

### Tryptophan fluorescence and quenching experiments

A luminescence spectrometer, Shimadzu RF5301 was used to measure the tryptophan fluorescence. Each peptide (2 μmol/L) was added to 1 mL HEPES buffer (10 mmol/L HEPES, 150 mmol/L NaCl) containing 0.1 mmol/L liposomes (pH 7.5), and the peptide/liposome mixture was allowed to interact at 25°C for 10 min. The fluorescence was excited at 280 nm, and the emission was scanned from 300 to 400 nm. The fluorescence spectrum of each peptide with liposomes/HEPES was collected after the deduction of the spectrum of liposomes/HEPES without peptide.

KI quenching experiments were carried out at an excitation wavelength of 280 nm. KI was added from a 2 mol/L stock solution to peptides in the absence or the presence of liposomes and the tested concentrations of KI were 0 mol/L, 0.02 mol/L, 0.04 mol/L, 0.06 mol/L and 0.08 mol/L. The experimental data were plotted according to the Stern-Volmer equation *F*_*0*_/*F* = 1 + *Ksv*[Q], where *F*_*0*_ and *F* are the fluorescence in the absence and the presence of a quencher at different concentrations [Q], respectively, and *Ksv* is the Stern-Volmer quenching constant (Eftink and Ghiron, 1976; Zhang et al., 1999).

## Abbreviations

AMPs: antimicrobial peptides
HPLC: high-performance liquid chromatography
LUV: large unilamellar vesicles
MHC: minimal hemolytic concentration
MIC: minimal inhibitory concentrations
